# A Compound Sensor for Simultaneous Measurement of Packing Density and Moisture Content of Silage

**DOI:** 10.3390/s18010073

**Published:** 2017-12-28

**Authors:** Delun Meng, Fanjia Meng, Wei Sun, Shuang Deng

**Affiliations:** 1 Key Laboratory on Modern Precision Agriculture System Integration Research, Ministry of Education, China Agricultural University, Beijing 100083, China; mdlun@126.com (D.M.); Dengs415@163.com (S.D.); 2 Agricultural Information Institute, Chinese Academy of Agricultural Sciences, Beijing 100081, China; sunwei01@caas.cn

**Keywords:** silage, packing density, moisture content, compound sensor, simultaneous measurement

## Abstract

Packing density and moisture content are important factors in investigating the ensiling quality. Low packing density is a major cause of loss of sugar content. The moisture content also plays a determinant role in biomass degradation. To comprehensively evaluate the ensiling quality, this study focused on developing a compound sensor. In it, moisture electrodes and strain gauges were embedded into an ASABE Standard small cone for the simultaneous measurements of the penetration resistance (PR) and moisture content (MC) of silage. In order to evaluate the performance of the designed sensor and the theoretical analysis being used, relevant calibration and validation tests were conducted. The determination coefficients are 0.996 and 0.992 for PR calibration and 0.934 for MC calibration. The validation indicated that this measurement technique could determine the packing density and moisture content of the silage simultaneously and eliminate the influence of the friction between the penetration shaft and silage. In this study, we not only design a compound sensor but also provide an alternative way to investigate the ensiling quality which would be useful for further silage research.

## 1. Introduction

Packing density and moisture content of silage are of great significance in guiding the fine processing of silage and improving its quality. A lower packing density means a higher porosity, which could result in more oxygen remaining in the silage. Excessive oxygen will cause carbohydrates reduction, protein denaturation and quality degradation during the aerobic phase [1,2,3,4,5,6,7,8,9]. In addition, the biomass moisture affects the activity of antioxidant bacteria and the degree of degradation of the biomass [1,10]. In general, to prevent the loss of dry matter and sugar content of silage, both factors should be optimally controlled.

The traditional method for determining the packing density of silage is to calculate its gross density from mass and volume. The disadvantage of this method is that it is unable to show density differences at specific sites. The γ-ray scanner is an effective tool to analyze the packing density in two dimensions and the relative error of measurement could be ±1% [11,12]. However, the γ-ray scanner is not widely applied due to its high cost and the potential danger to health [2,3,4]. In recent years, the penetrometer technique has been used to measure the packing density of silage because it is straightforward to calibrate and can provide reliable data [13]. In many penetrometer designs [1,10], the force sensor was placed on the upper end of the penetration shaft. Given that silage was a complex porous elastoplastic medium, the penetration shaft would be extruded laterally by silage in the penetration process. Therefore, penetration resistance (PR) obtained by these penetrometers was generally referred to as a superposition of cone resistance and the friction force between the penetration shaft and the tested material [14]. To improve the measuring accuracy of the penetrometer, Y. Sun et al. developed a mathematical filter to correct the friction-induced error of PR measurements [14,15]. For the MC measurement of silage, the stoving method and the infrared method have been employed [16,17]. The stoving method has not met the production process’s requirements because of its time-consumption and sample destruction. For the infrared method, the configuration of the sensor was complicated and not robust. Silage, like soil, is a complex porous elastoplastic medium and PR is not only strongly dependent on packing density but also on water content [18]. Most instruments applying the above-mentioned measurement methods are designed to obtain a specified parameter and are unable to accurately evaluate the ensiling quality. In this study, a novel compound sensor was designed to measure the PR and MC of silage simultaneously and calibrated in the laboratory. Validation tests were conducted to verify its feasibility and ascertain the correlation between the PR and MC of silage.

## 2. Compound Sensor Design

**Figure 1:**
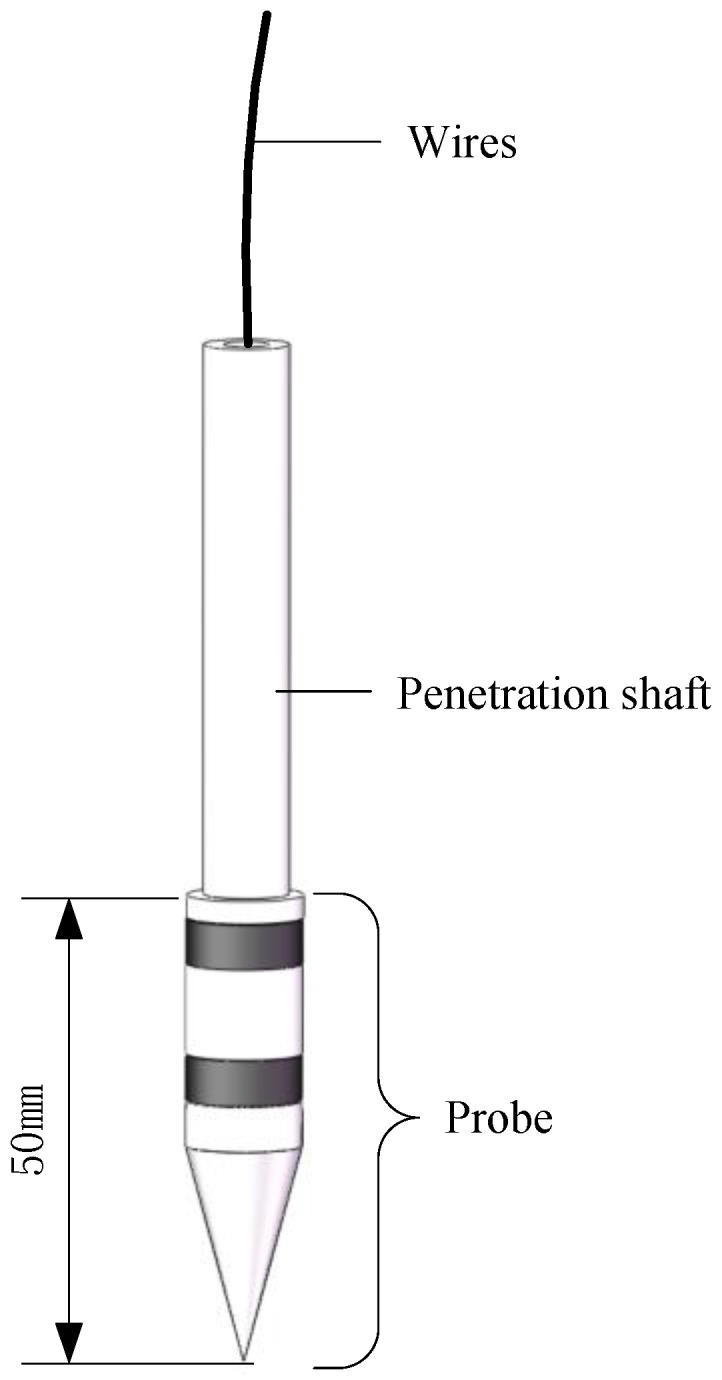
Diagram of the compound sensor.

The compound sensor, shown in a schematic diagram (Figure 1), consists of a probe, a penetration shaft and wires. The probe and the penetration shaft are made of nickel chromium alloy material and designed strictly based on the ASABE Standard [19]. The angle of the cone is 30 degrees, the diameter of the cone is 12.83 mm and the diameter of the shaft is 9.53 mm. In addition, the wire connects the probe and the measurement circuits through the penetration shaft.

**Figure 2:**
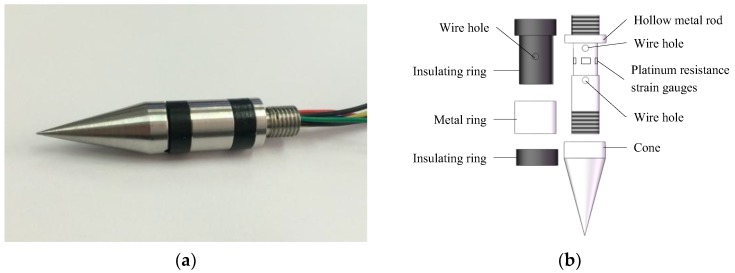
The probe of the compound sensor. (**a**) A photo of the probe; (**b**) Detailed structure of the probe.

**Figure 3:**
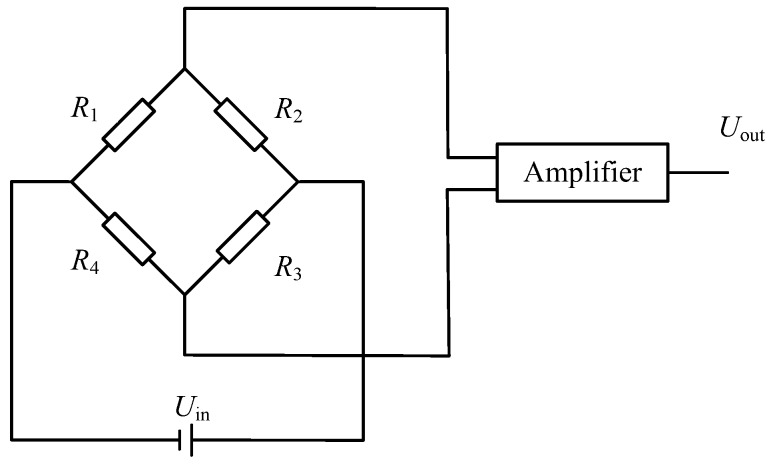
Diagram of the Wheatstone bridge circuit.

A photo of the probe of the compound sensor is shown in Figure 2a. The measurement principle of PR is based on the fact that the resistance of a metal conductor varies with the magnitude of its mechanical deformation, that is, the resistance-strain effect. In order to eliminate the influence of the friction on the shaft, resistance-strain gauges are embedded into the probe and Figure 2b illustrates the structure. The hollow metal rod was regarded as an elastic body and four platinum resistance-strain gauges (350 Ω) are pasted on the outer surface of it. Wires connect the platinum resistance-strain gauges to form a Wheatstone bridge circuit, as shown in Figure 3.

In the Wheatstone bridge circuit, each arm of the bridge contains a platinum resistance strain gauge. When the cone is compressed, the hollow metal rod will deform. Based on the resistance-strain effect of metal, the resistance of the strain gauge would change according to the mechanical deformation. The formula for calculating the voltage output *U*_out_ of the bridge is

(1) $$U_{\mathrm{out}}=A\frac{R_{1}R_{3}-R_{2}R_{4}}{\left(R_{1}+R_{2}\right){(R}_{3}+R_{4})}U_{\mathrm{in}}$$

where *U*_out_ represents the output voltage of the bridge and *U*_in_ represents the input voltage of the bridge. *R*_1_, *R*_2_, *R*_3_ and *R*_4_ refer to the resistance values of the bridge arm respectively and *A* is the amplification factor. The measurement force range of the compound sensor is 0–1 kN and the output signal of the bridge corresponds to 0–2.5 V.

**Figure 4:**
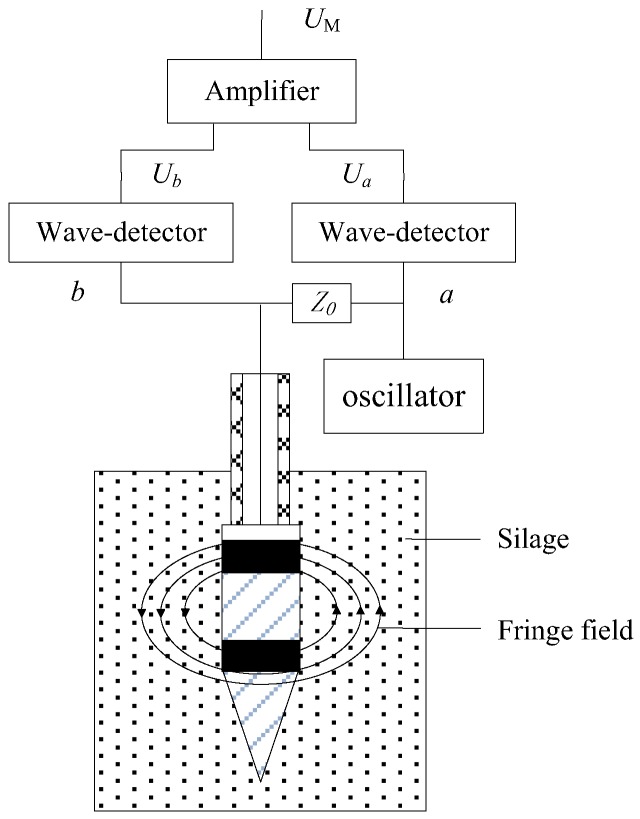
Schematic diagram of the compound sensor for moisture content measurement.

The measurement principle of MC is based on the dielectric theory, according to which the relative dielectric permittivity of water is 81, far greater than that of the drying biological materials (ε_r-wood_ ≈ 3) and that of air (ε_r-air_ = 1) [1]. The water content of biological materials directly affects the relative dielectric permittivity. Therefore, the MC of biomaterials can be indirectly obtained by measuring the relative dielectric permittivity. Thus, the moisture content of silage is measured by the frequency domain method [20,21,22,23] in this study and the working principle is shown in Figure 4.

The metal ring and cone, separated by two insulating rings, acted as the moisture electrodes of the compound sensor. A segment of coaxial line cable through the center of the penetration shaft connected both electrodes to an oscillator (100 MHz). Based on the measurement principle of the moisture content, the characteristic impedance of the probe depended on the fringe-effect field across the two electrodes and the relative dielectric permittivity of the silage, which varied with the ratio of water. Therefore, there is a mapping relation between the characteristic impedance of the probe and silage moisture content. *Z*_P_ (Ω), standing for the electric impedance of the probe, can be determined from Equation (2)

(2) $$Z_{P}=\frac{Z_{0}}{U_{a}-U_{b}}U_{b}$$

where *Z*_0_ (Ω) is called balance impedance and *U*_a_ and *U*_b_ are the output signals of each wave detector.

When the length of the transmission line is λ/4 (λ is the test frequency wavelength), the MC signal, *U*_M_, can be calculated by

(3) $$U_{M}=U_{a}-U_{b}=2B\frac{Z_{P}-Z_{0}}{Z_{P}+Z_{0}}$$

where *B* represents the amplitude of the excitation signal.

## 3. Results and Discussion

### 3.1. Force Calibration

**Figure 5:**
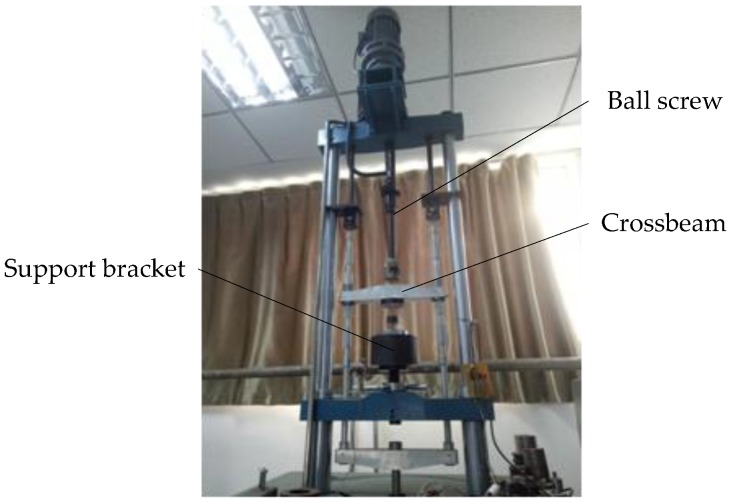
A photo of the force-testing machine.

**Figure 6:**
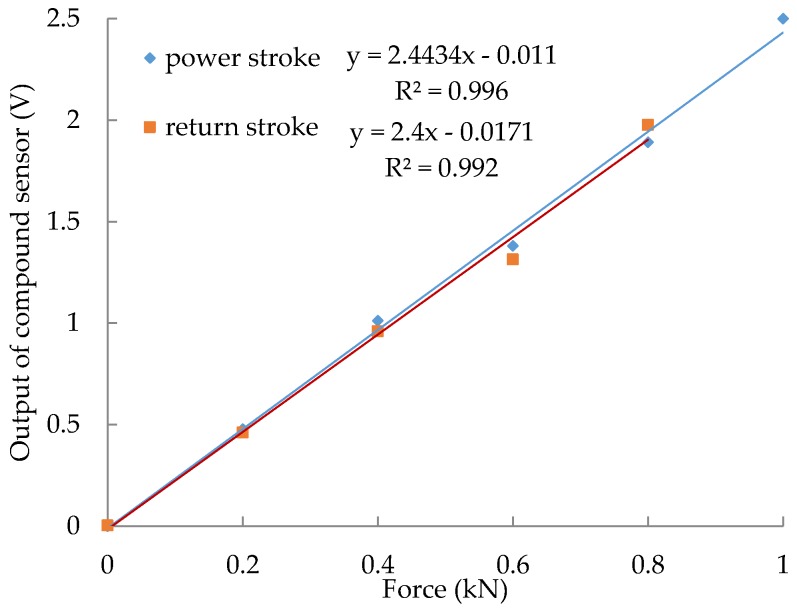
The force calibration results.

The intention of the force calibration is to establish the relationship between the output signal of the force measuring circuit and the force applied to the compound sensor. To create a reference calibration, a force-testing machine (DWM-10, 0~10 kN, 0.01%, Suzhou Longsheng Testing Equipment Co. Ltd., Suzhou, China) was utilized to apply force to the compound sensor, as shown in Figure 5. The compound sensor was bolted to the support bracket. As the crossbeam, which was driven by a ball screw, moved down or up, the weights were loaded or unloaded steadily. The calibration includes two processes: power stroke and return stroke. The calibration result (Figure 6) shows that, in each process, there is a linear equation with a determination coefficient (R^2^) of 0.99 relating the output of the compound sensor to force values within a range of 0–1 kN.

### 3.2. Calibration of Moisture Content

**Figure 7:**
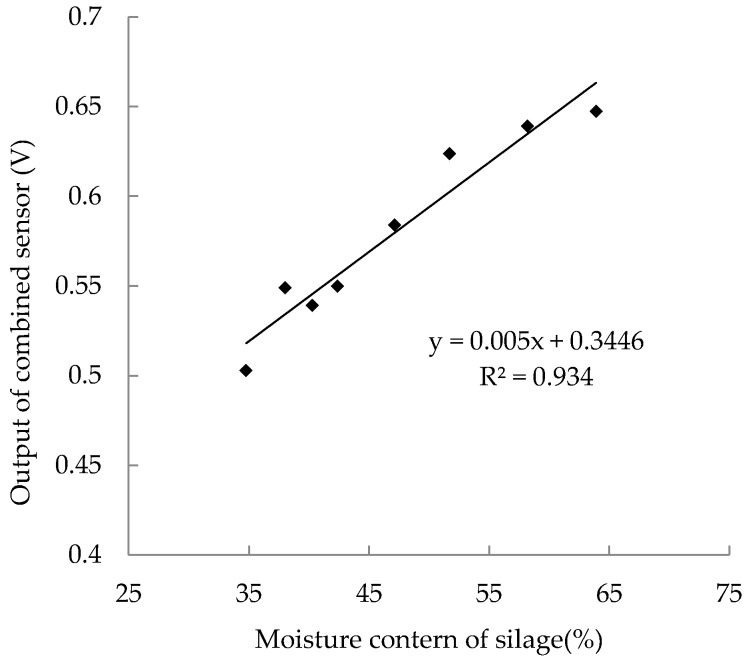
The calibration of moisture content results.

The silage material for calibration was chopped maize with moisture content of 63.9%. Before the calibration, eight silage samples were dried at 105 °C for different degrees and filled into cylinders (inner radius: 200 mm, height: 500 mm) with a density of 0.8 g/cm^3^ respectively. To ensure that the density was distributed homogeneously, the chopped maize samples were pressured into this cylinder by a material-testing machine (Zwick 1445, Zwick GmbH & Co. KG, Ulm, Germany) layer by layer. Each sample was sampled three times and the arithmetic average value of the output voltage was recorded. After the measurements, the silage samples were weighed and oven-dried for 24 h at 105 °C, then weighed again and their moisture content was calculated based on the mass of wet and dry samples. The calibration result (Figure 7) shows a linear relationship between the measured MC values and the output signals of the compound sensor within a range of 34.8~63.9% and the correlation is approximated by a linear calibration equation with the determination coefficients (R^2^) of 0.934.

### 3.3. Validation Test

**Figure 8:**
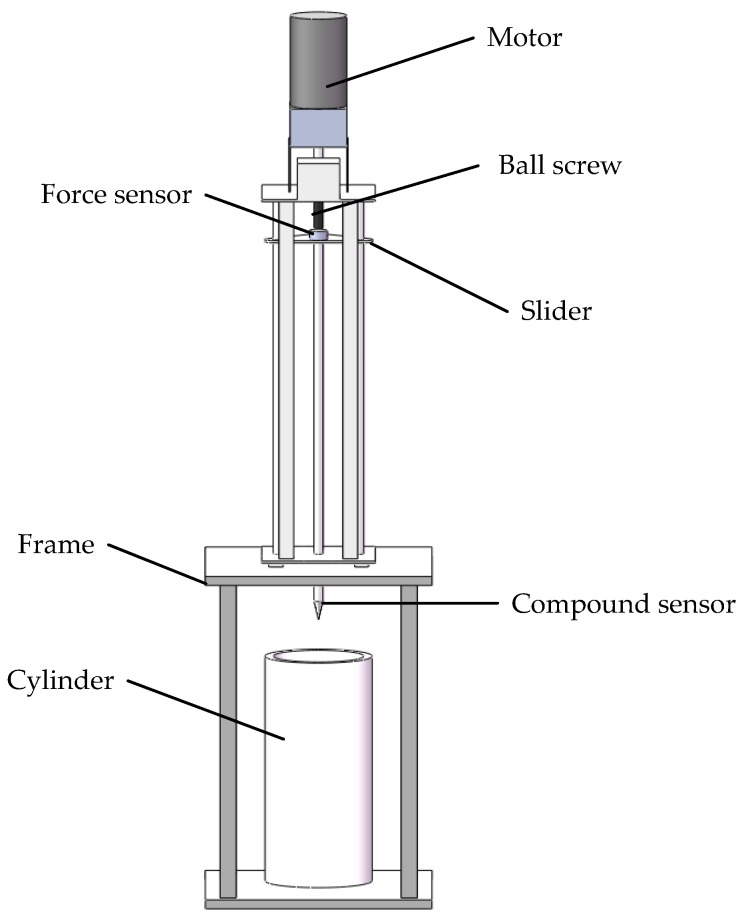
Diagram of the validation system.

A validation system, which includes a motor-operated penetrometer, a frame and a cylinder, is shown in Figure 8. According to the ASABE Standard, to ensure the penetration speed of the compound sensor is stable at 30 mm/s and to improve the operation and control accuracy during the penetration process, the ball screw was selected as the transmission part. The ball screw is driven by a motor (100 W, 1800 r/min, permanent magnet direct current motor, Shanghai Weiting Electric Co. Ltd., Shanghai, China) through a reduction gearbox (reduction ratio: 5:1). A slider is motivated by the ball screw to slide along the axis, thereby converting the rotation of the motor to vertical motion. The compound sensor is installed below the slider and a force sensor (BK-4D, 0~1000 N, 0.5%, CAAA, Beijing, China) is installed at the upper end of the penetration shaft. When the slider moves downward and the cone penetrates the testing silage, the validation system can synchronously obtain the pressure measurement data of these two sensors in real time. The vertical travel of the penetrometer cone could be adjusted from 100 mm to 700 mm and an encoder (500 p/r), acting as a transducer, was set up to output the depth signal of the compound sensor. When the cone reached the predetermined penetration depth or when the PR value exceeded 1000 N, the DC motor would automatically reverse. A micro-controller (MSP430F149, TI, Dallas, TX, USA) was used to log data and coordinate the entire measurement in sequence.

**Figure 9:**
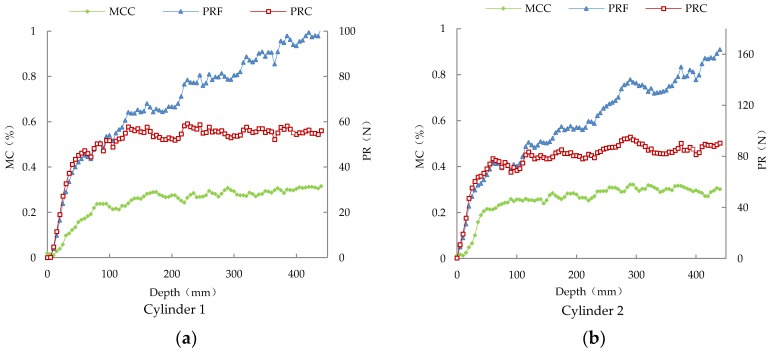

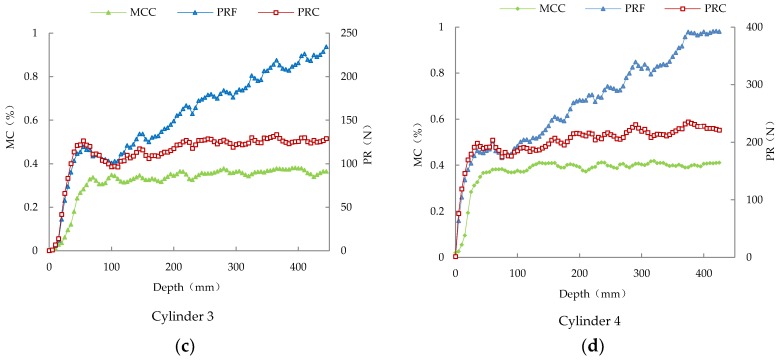
Dynamic measurement results of the compound sensor of Cylinder 1 to Cylinder 4.

According to the methods described in Section 3.2, 12 cylinders of silage were prepared and the sample information is presented in Table 1. The measurement results of four samples (Cylinder 1 to Cylinder 4) are shown in Figure 9. Within the first insertion where depths are less than 50 mm, the MC signal of the compound sensor (MCC) and the PR signal of the compound sensor (PRC) show great increase. This is due to the fact that, in the initial insertion, the two electrodes of the probe are not fully embedded in the silage and the action and reaction between the cone and the silage do not reach steady state. When insertion depths are greater than 50 mm, the curves of both signals of the compound sensor tend to be horizontal. This shows that MCC keeps close track of PRC, indicating that the compound sensor can determine the packing density and moisture content of the silage simultaneously.

As can be seen from Figure 9, in the initial insertion, the penetration resistance signals of the force sensor (PRF) are basically the same as PRC. Then after the probe was completely embedded in the silage, PRF profiles showed a linear relation to the penetration depth or the contacted area between the shaft and the silage and PRC profiles reached a plateau. For the homogeneous distribution of density in all the cylinders, packing density would change little by depth and this agrees with PR profiles measured with the compound sensor. This shows that the compound sensor designed in this study can effectively eliminate the influence of the friction between the penetration shaft and silage, improving the measurement accuracy.

**Figure 10:**
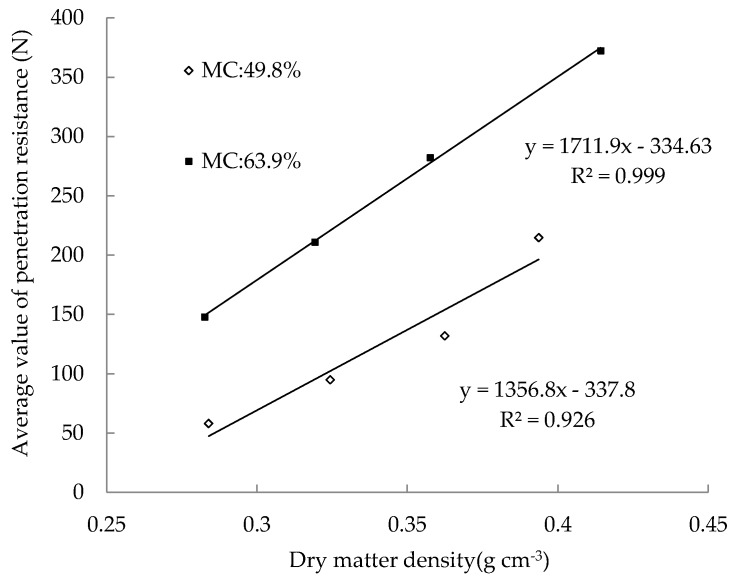
Average value of penetration resistance of silages with two different moisture contents.

**Figure 11:**
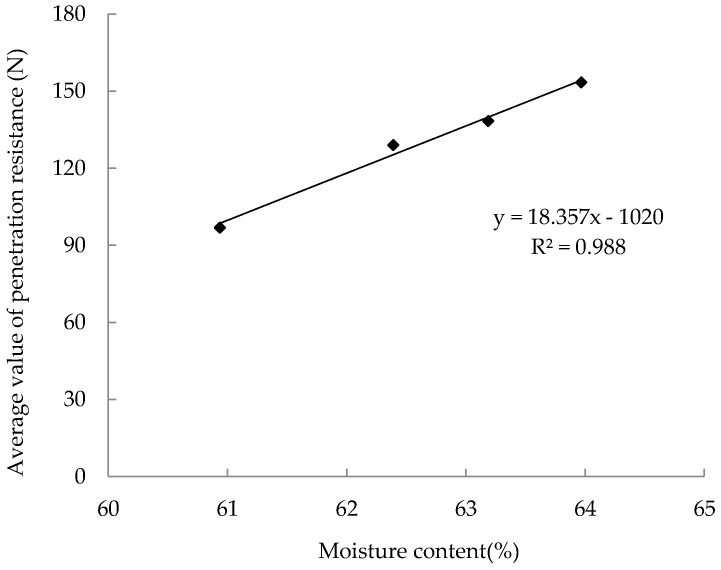
Average value of penetration resistance and volumetric moisture content of silages with the same dry matter density.

Since the 12 samples were evenly packed into cylinders, the average value of the PR at different depths is taken as the PR values of the sample. For samples from Cylinder1 to Cylinder8, the correlation between the dry matter density and the PR value is shown in Figure 10. For the silage samples with the same MC, the PR values increase linearly with the dry matter density and correlations are approximated by tow linear calibration equations with the determination coefficients (R^2^) of 0.999 and 0.926 respectively. On the other hand, for samples with the same dry matter density, the PR of samples with high MC is greater than those with low MC. As expected in Figure 11, there is a linear equation with R^2^ of 0.988 relating the PR and the MC of four samples (Cylinder 9 to Cylinder 12). It demonstrates that, under the same dry matter density condition, the PR of the silage increases linearly with the MC of the sample.

## 4. Conclusions

A compound sensor, designed based on ASABE standards, was developed as a novel technique for evaluating the quality of silage. In this compound sensor, the moisture electrode and strain gauges were embedded in an ASABE Standard small cone, which made the compound sensor capable of measuring the packing density and moisture content of silage simultaneously. From the results of relevant calibrations and validation, the following conclusions can be drawn: (1) The packing density and moisture content of the silage are linearly related to the outputs of the compound sensor and both the determination coefficients are greater than 0.93. This indicates that the designed sensor could obtain the packing density and moisture content of the silage accurately. (2) The compound sensor could determine the packing density and moisture content of the silage simultaneously and eliminate the influence of the friction force between the penetration shaft and the surrounding silage. (3) For the samples with the same moisture content, the packing density increases with the increase of dry matter density. The packing density of the samples with the same dry matter density increases in accordance with the moisture content of these samples.

## Figures and Tables

**Table 1:** Sample Information.

	Gravimetric Moisture Content (%)	Mass (g)	Volume (cm^3^)	Dry Matter Density (g/cm^3^)
Cylinder 1	49.8	9150	15,700	0.284
Cylinder 2		10,150		0.324
Cylinder 3		11,151		0.362
Cylinder 4		12,150		0.394
Cylinder 5	63.9	12,650		0.283
Cylinder 6		14,150		0.319
Cylinder 7		15,650		0.358
Cylinder 8		17,150		0.414
Cylinder 9	60.9	12,022		0.299
Cylinder 10	62.4	12,487		
Cylinder 11	63.2	12,756		
Cylinder 12	64.0	13,033		
